# Modeling and validation of drug release kinetics using hybrid method for prediction of drug efficiency and novel formulations

**DOI:** 10.3389/fchem.2024.1395359

**Published:** 2024-06-21

**Authors:** Saad M. Alshahrani, Hadil Faris Alotaibi, Mohammed Alqarni

**Affiliations:** ^1^ Department of Pharmaceutics, College of Pharmacy, Prince Sattam Bin Abdulaziz University, Al-Kharj, Saudi Arabia; ^2^ Department of Pharmaceutical Sciences, College of Pharmacy, Princess Nourah Bint AbdulRahman University, Riyadh, Saudi Arabia; ^3^ Department of Pharmaceutical chemistry, College of Pharmacy, Taif University, Taif, Saudi Arabia

**Keywords:** durg delivery, decision tree regression, passive aggressive regression, quadratic polynomial regression, modeling

## Abstract

This paper presents a thorough examination for drug release from a polymeric matrix to improve understanding of drug release behavior for tissue regeneration. A comprehensive model was developed utilizing mass transfer and machine learning (ML). In the machine learning section, three distinct regression models, namely, Decision Tree Regression (DTR), Passive Aggressive Regression (PAR), and Quadratic Polynomial Regression (QPR) applied to a comprehensive dataset of drug release. The dataset includes *r*(m) and *z*(m) inputs, with corresponding concentration of solute in the matrix (C) as response. The primary objective is to assess and compare the predictive performance of these models in finding the correlation between input parameters and chemical concentrations. The hyper-parameter optimization process is executed using Sequential Model-Based Optimization (SMBO), ensuring the robustness of the models in handling the complexity of the controlled drug release. The Decision Tree Regression model exhibits outstanding predictive accuracy, with an R^2^ score of 0.99887, RMSE of 9.0092E-06, MAE of 3.51486E-06, and a Max Error of 6.87000E-05. This exceptional performance underscores the model’s capability to discern intricate patterns within the drug release dataset. The Passive Aggressive Regression model, while displaying a slightly lower R^2^ score of 0.94652, demonstrates commendable predictive capabilities with an RMSE of 6.0438E-05, MAE of 4.82782E-05, and a Max Error of 2.36600E-04. The model’s effectiveness in capturing non-linear relationships within the dataset is evident. The Quadratic Polynomial Regression model, designed to accommodate quadratic relationships, yields a noteworthy R^2^ score of 0.95382, along with an RMSE of 5.6655E-05, MAE of 4.49198E-05, and a Max Error of 1.86375E-04. These results affirm the model’s proficiency in capturing the inherent complexities of the drug release system.

## 1 Introduction

Efficient delivery of therapeutic agents to the desired site has been a subject of research owing to the importance of this method in cancer treatment. Drugs might reach other tissues and damage them, while low dosage of drug could reach the cancer cells for treatment. Therefore, the design of targeted drug delivery systems would be of fundamental importance for cancer effective treatment (Kandula et al., 2023; Chen et al., 2024; Lu et al., 2024; Sameer Khan et al., 2024). Drug can be loaded into various carriers such as polymeric nanoparticles and reach the target cells, while its release can be triggered by various means such as pH or temperature change (Ali et al., 2023).

Modeling and computation of drug release from carriers can be utilized for design and optimization of drug delivery systems based on polymeric carriers. Some mathematical models have been developed to simulate mass transfer in polymeric-based drug release (González-Garcinuño et al., 2023; Kubinski et al., 2023; Carr and Pontrelli, 2024). Usually, molecular diffusion is the main mechanism that happens in polymeric-based drug delivery systems where the drug molecules diffuse through the porous structure of polymeric carrier. Some parameters such as pore structure of carrier, molecular interaction, temperature, and pH can affect the release rate of drug molecules. On the other hand, machine learning models can be used for simulation of drug release from polymeric carriers. The method is based upon collection of datasets and building models via appropriate algorithms. This method is indeed fast and possesses higher performance in terms of fitting accuracy.

Machine learning (ML) techniques have shown great potential in the field of drug development by enabling accurate forecasting of drug solubility and density (Abdelbasset et al., 2022; Almehizia et al., 2023). These techniques have the capability to evaluate large amounts of data and extract meaningful patterns and relationships that can be utilized for predictions (Jovel and Greiner, 2021). This paper provides a thorough analysis of three distinct regression models, namely, Decision Tree Regression (DTR), Passive Aggressive Regression (PAR), and Quadratic Polynomial Regression (QPR). These models were carefully evaluated using a comprehensive dataset in the field of drug release from a porous polymeric carrier. The hyper-parameter optimization process is executed using Sequential Model-Based Optimization (SMBO).

Decision Tree Regression is a versatile algorithm that can be utilized in a wide range of regression tasks. Careful tuning of hyperparameters is essential to prevent overfitting and ensure optimal model performance (Talekar and Agrawal, 2020). Passive Aggressive Regression offers a flexible and adaptive approach to regression tasks, particularly in situations where data arrives sequentially or in a streaming fashion (Crammer et al., 2006). Also, Quadratic Polynomial Regression is a valuable tool for capturing quadratic relationships in the data. Careful consideration of model complexity and potential overfitting is crucial for obtaining reliable and meaningful results (Yao and Müller, 2010).

By systematically evaluating Decision Tree Regression (DTR), Passive Aggressive Regression (PAR), and Quadratic Polynomial Regression (QPR) models on a dataset comprising over 15,000 data samples, the study provides valuable insights into the strengths and limitations of each model. The incorporation of Sequential Model-Based Optimization (SMBO) for hyper-parameter tuning enhances the robustness of the models, highlighting the significance of thoughtful parameter optimization.

## 2 Problem statement

**FIGURE 1:**
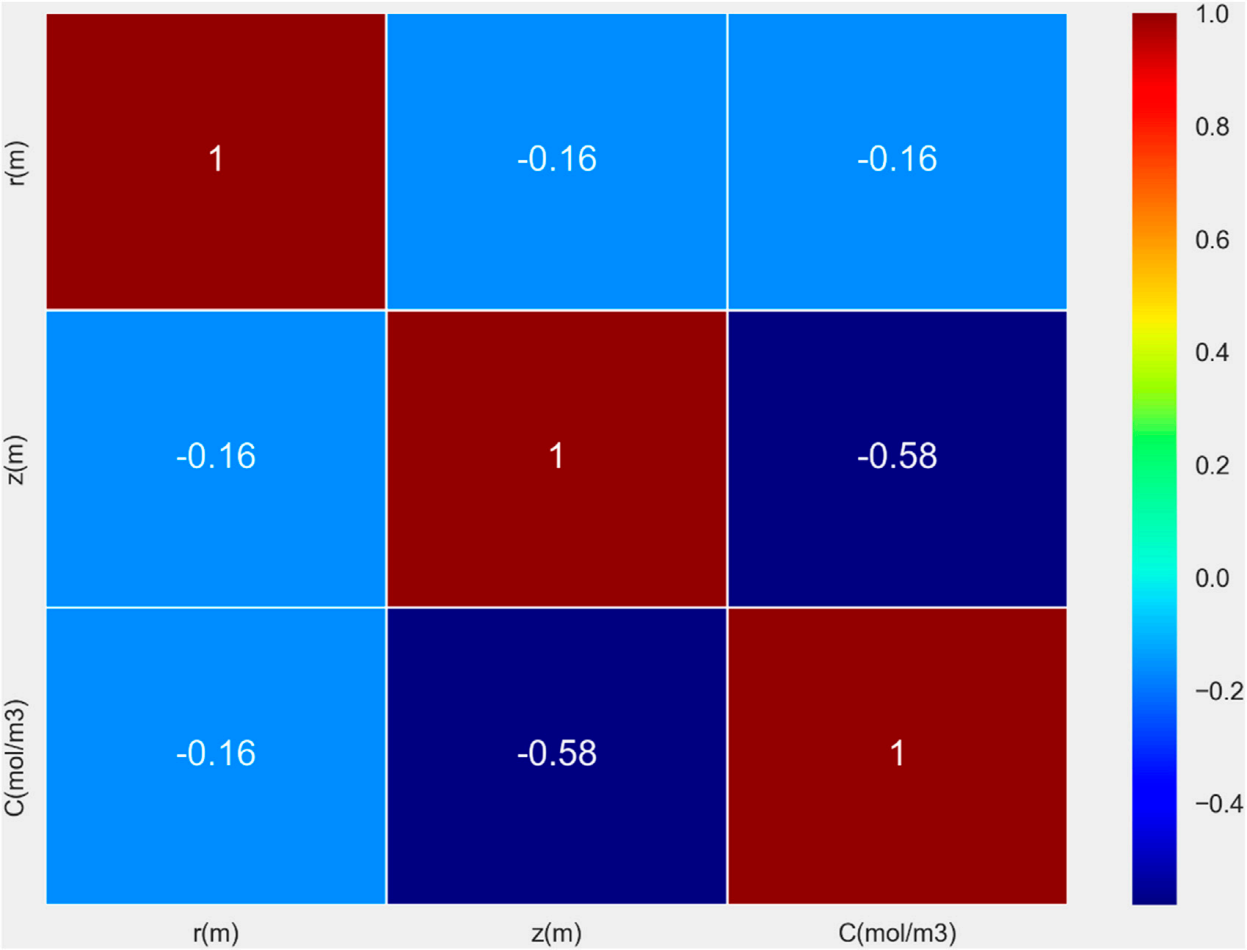
Correlation heatmap for drug release dataset.

This research dataset consists of more than 15,000 data points, incorporating three key variables: *r* measured in meters, *z* also in meters, and chemical concentration *C* expressed in mol/m^3^. The data have been collected from a CFD (Computational Fluid Dynamics) simulation of drug-loaded polymeric matrix. The CFD was utilized to numerically solve time-dependent mass balance per species (COMSOL, 2008) and the generated data was used for building the machine learning models. The correlation heatmap between variables is shown in Figure 1. This step was done as the preliminary data visualization to see how data vary in the domain of drug delivery system.

The z-score, or standard score, is a statistical measure widely employed for outlier detection in various studies, including the present research. When conducting outlier analysis, the z-score is a useful metric that provides a standardized representation of the deviation of a data point from the mean of the dataset. It measures the distance from a data point to the mean in standard deviation units.

The expression for determining the z-score of a data point *X* within a dataset having a mean of *μ* and a standard deviation of *σ* is articulated as follows (Anusha et al., 2019):

$$Z=\frac{\left(X-\mu \right)}{\sigma }$$

In this context, *Z* signifies the z-score of the data point, *X* represents the individual data value, *μ* is indicative of the mean within the dataset, and *σ* denotes the standard deviation.

A high absolute z-score indicates that the data point is far from the mean and is considered a potential outlier. The threshold for identifying outliers using z-scores is often set empirically; commonly, a z-score beyond 2 or 3 standard deviations is considered indicative of an outlier.

**FIGURE 2:**
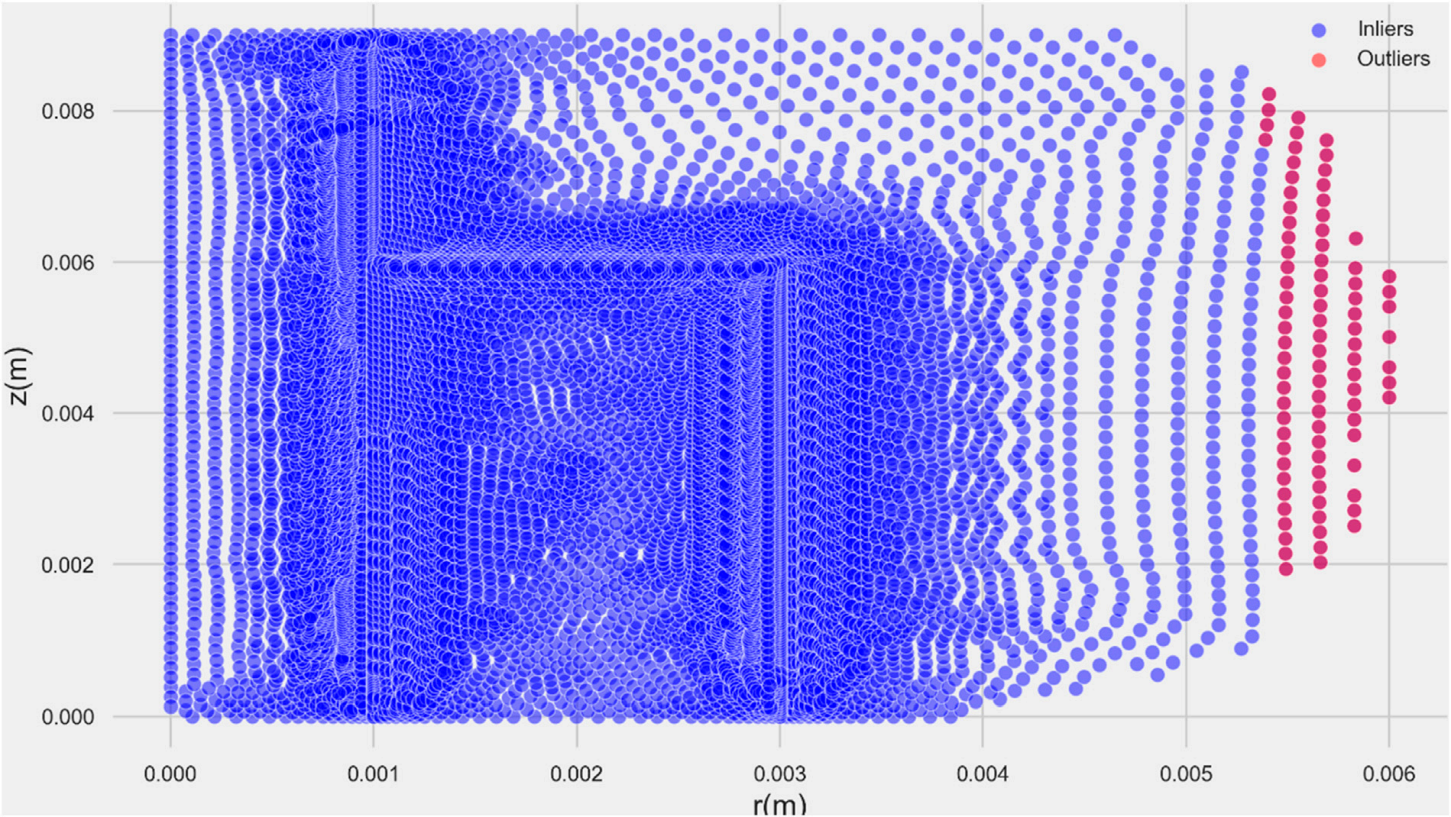
Z-Score plot analysis.

In the specific context of this study, the z-score method has been employed for outlier detection. By calculating z-scores for the relevant variables or features, the study aims to identify data points that exhibit significant deviations from the norm, facilitating a robust analysis of the dataset and ensuring the reliability of the research findings. The result of z-score analyses is shown in Figure 2.

## 3 Method of computing

### 3.1 Sequential Model-Based Optimization (SMBO)

Sequential Model-Based Optimization (SMBO) emerges as a powerful strategy for optimizing hyperparameters within the domain of machine learning. It seamlessly integrates elements of Bayesian optimization and model-driven reasoning to systematically navigate the hyperparameter landscape, identifying optimal configurations for a given machine learning model (Croppi, 2021).

Hyperparameters, distinct from model parameters learned during training, constitute pre-defined configuration settings governing a model’s behavior and performance. The paramount goal of hyperparameter tuning is to pinpoint the most favorable values for these settings, significantly influencing the overall performance of the model.

SMBO is a technique that utilizes Bayesian optimization principles to optimize a given objective function. The core idea behind SMBO is to iteratively assess and update a surrogate model, which approximates the true objective function. This surrogate model guides the optimization process by estimating the objective function based on evaluated hyperparameter configurations (Lacoste et al., 2014).

At each iteration, SMBO selects the subsequent hyperparameter configuration for evaluation, striking a balance between exploration and exploitation. This decision is informed by an acquisition function denoted as *a(x)*, gauging the utility of evaluating a specific configuration *x* based on predictions from the surrogate model. The acquisition function incorporates both the predicted performance $\hat{f}\left(x\right)$ and uncertainty $\sigma \left(x\right)$ of the surrogate model (Croppi, 2021):

$$a\left(x\right)=\alpha \left(x\right)\cdot \mu \left(x\right)+\beta \left(x\right)\cdot \sigma \left(x\right)$$

Here, $\mu \left(x\right)$ signifies the anticipated performance of the surrogate model, while $\alpha \left(x\right)$ and $\beta \left(x\right)$ are weighting functions regulating the trade-off between exploitation and exploration. The choice of acquisition functions depends on specific optimization objectives (Tran et al., 2019).

To establish the surrogate model, SMBO initiates with a random sample of hyperparameter configurations, refining and updating them iteratively based on the acquisition function until a stopping criterion is met.

SMBO’s merits in hyperparameter tuning include its efficiency in exploring hyperparameter space, capacity to capture intricate interactions between hyperparameters, automated configuration process, and adaptability to diverse hyperparameters and machine learning algorithms.

### 3.2 Decision Tree Regression model (DTR)

Decision Tree Regression (DTR) stands out as a potent tool in the realm of machine learning, serving the purpose of predictive modeling and regression analysis. Differing from its classification equivalent, Decision Tree Classification, DTR has a distinct focus on forecasting continuous values. Its operation involves the iterative division of the dataset into subsets based on the features’ values, leading to the formation of a tree-like arrangement of decision nodes (Kotsiantis, 2013).

Consider *X* as the input feature matrix comprising *n* samples and *m* features, while *y* represents the corresponding target variable. The Decision Tree Regression model can be expressed as (Rokach et al., 2005; Olson et al., 2020):

$$\hat{y}=\sum_{i=1}^{N}c_{i}\cdot I\left(x\in R_{i}\right)$$

Here, $\hat{y}$ signifies the predicted output, *N* signifies the quantity of leaf nodes in the tree, $c_{i}$ stands for the constant value associated with the *i*-th leaf, $I\left(x\in R_{i}\right)$ denotes an indicator function that equals *1* if *x* belongs to the *i*-th region $R_{i}$ and 0 otherwise.

The objective of the model is to identify optimal values for the parameters $c_{i}$ and the corresponding regions $R_{i}$ in order to minimize the sum of squared differences between the model predicted values and the expected (true) target values.

**FIGURE 3:**
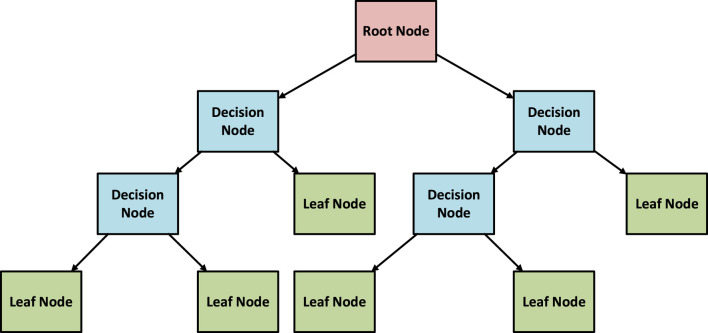
Structure of DTR model.

The structure of DTR model is displayed in Figure 3. In the training procedure, the dataset undergoes iterative division into subsets by leveraging feature thresholds. The algorithm meticulously picks the feature and its associated threshold, aiming to minimize the mean squared error (MSE) concerning predictions within each subset. The recursive partitioning persists until a predetermined stopping condition is met, whether it involves reaching a maximum tree depth or satisfying a minimum threshold of samples per leaf (Quinlan, 1986; Suthaharan et al., 2016). Advantages of Decision Tree Regression can be summarized in following items (Bertsimas et al., 2017):

1. Non-linearity Handling: DTR excels in taking complicated non-linear associations between input parameters and the response variable, rendering it well-suited for handling complex datasets.
2. Interpretability: Decision trees are inherently interpretable, allowing users to easily understand and visualize the decision-making process.
3. Robustness to Outliers: DTR is robust to outliers as it makes decisions based on splits, rather than relying on the mean or median.

### 3.3 Passive Aggressive Regression (PAR)

Passive Aggressive Regression (PAR) is a type of online learning algorithm used for regression tasks. It is particularly suitable for scenarios where data streams in real-time, and the model needs to adapt and update its parameters continuously. The “Passive Aggressive” name stems from its aggressive updating strategy when making incorrect predictions and passive behavior when predictions are correct (Salas et al., 2015).

The Passive Aggressive Regression model is defined by the following update rule (Crammer et al., 2006):

$$w^{\left(t+1\right)}=\arg\min_{w}\left\{\frac{1}{2}\left|w\right.-w^{\left(t\right)}{|}^{2}+C\max\left(0,\left|y^{\left(t\right)}-w^{T}x^{\left(t\right)}\right|\right)\right\}$$

Here w indicates the weight vector, $w^{\left(t\right)}$ denotes the weight vector at time step *t*, *C* stands for the regularization parameter, $y^{\left(t\right)}$ represents the true target at time step *t*, $x^{\left(t\right)}$ is the input feature vector at time step *t*, $\left|\cdot \right|$ denotes the Euclidean norm, and $\max\left(0,\cdot \right)$ is the hinge loss function.

### 3.4 Quadratic Polynomial Regression model (QPR)

QPR has been known as a polynomial regressive technique that extends the linear regression technique to find quadratic correlations between the input features and the response parameters. Unlike simple linear models which consider a linear relationship, QPR accommodates more complex curvilinear patterns in the data (Heiberger and Neuwirth, 2009; Yao and Müller, 2010; Almehizia et al., 2023).

Let *X* represent the input feature matrix with *n* data points and *m* features, and *y* be the corresponding target variable. The QPR model is defined by the equation (Yao and Müller, 2010; Almehizia et al., 2023):

$$\hat{y}=\beta_{0}+\beta_{1}x+\beta_{2}x^{2}$$

Where, $\hat{y}$ represents the predicted output, $\beta_{0}$ is the intercept term, $\beta_{1}$ stands for the coefficient associated with the linear term, $\beta_{2}$ represents the coefficient associated with the quadratic term, and x denotes the input feature.

## 4 Results and discussion

The evaluation of the Decision Tree Regression (DTR), Passive Aggressive Regression (PAR), and Quadratic Polynomial Regression (QPR) models was conducted on a dataset comprising more than 15,000 data points, with input parameters represented by *r*(m) and *z*(m) coordinates, and the output parameter denoted by concentration (C) in mol/m³. The models underwent hyper-parameter optimization using Sequential Model-Based Optimization (SMBO). Table 1 presents a summary of the numeric results obtained from the assessment conducted. This table provides a concise overview of the key metrics and performance measures obtained from the evaluation of the regression models.

**TABLE 1:** Final metrics of the optimized models.

Model	R^2^ score	RMSE	MAE	Max error
DTR	0.99887	9.0092E-06	3.51486E-06	6.87000E-05
PAR	0.94652	6.0438E-05	4.82782E-05	2.36600E-04
QPR	0.95382	5.6655E-05	4.49198E-05	1.86375E-04

**FIGURE 4:**
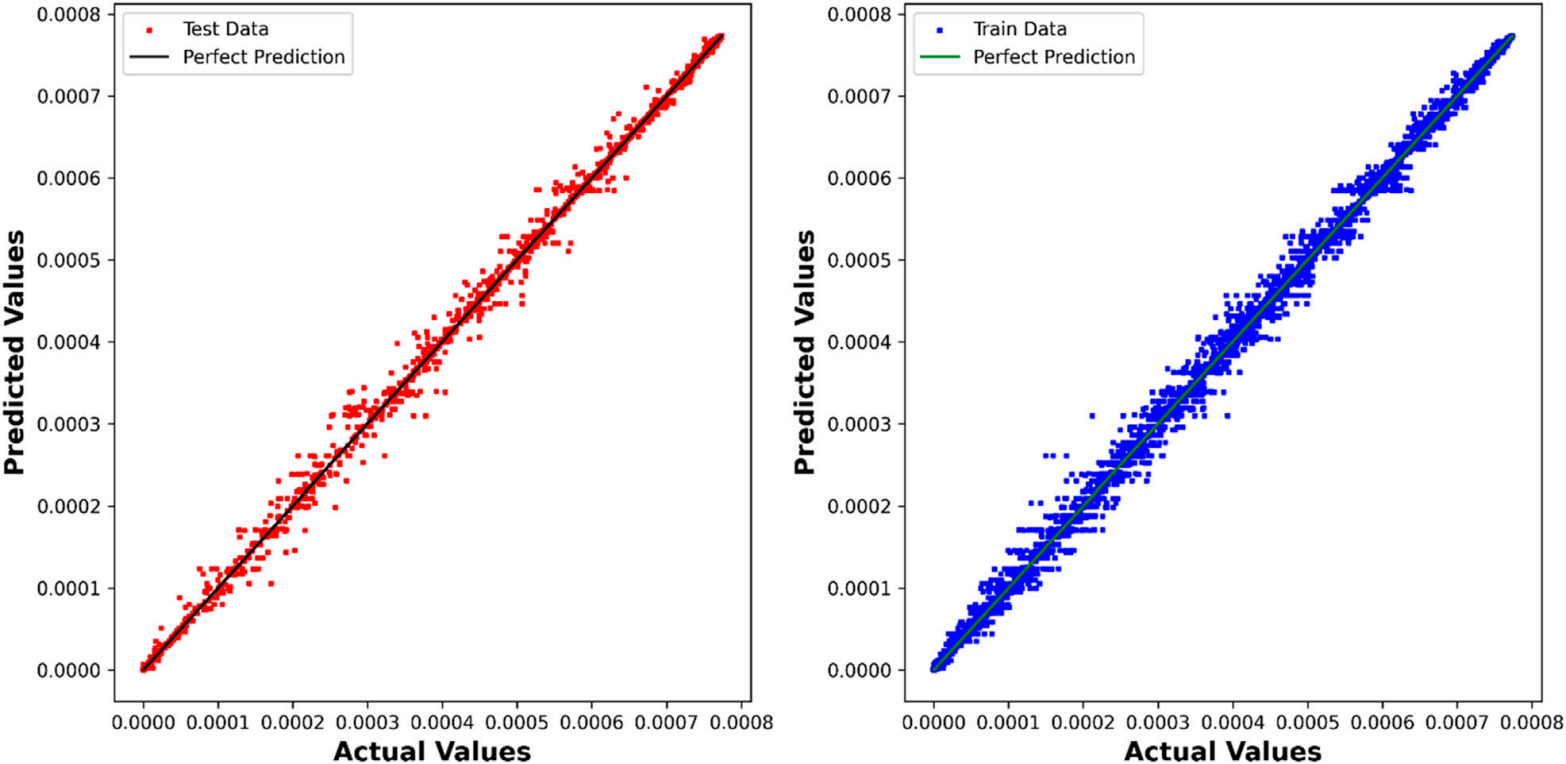
DTR model: Predicted values compared to True values.

The DTR model demonstrates outstanding predictive accuracy, reflected in an impressive R^2^ score of 0.99887, underscoring its capability to discern intricate patterns within the dataset. The negligible RMSE, MAE, and Max Error values further emphasize the precision and reliability of the model in predicting chemical concentrations. Figure 4 showcases a visual comparison between the model predicted values and the true values using the Decision Tree Regression (DTR) model.

**FIGURE 5:**
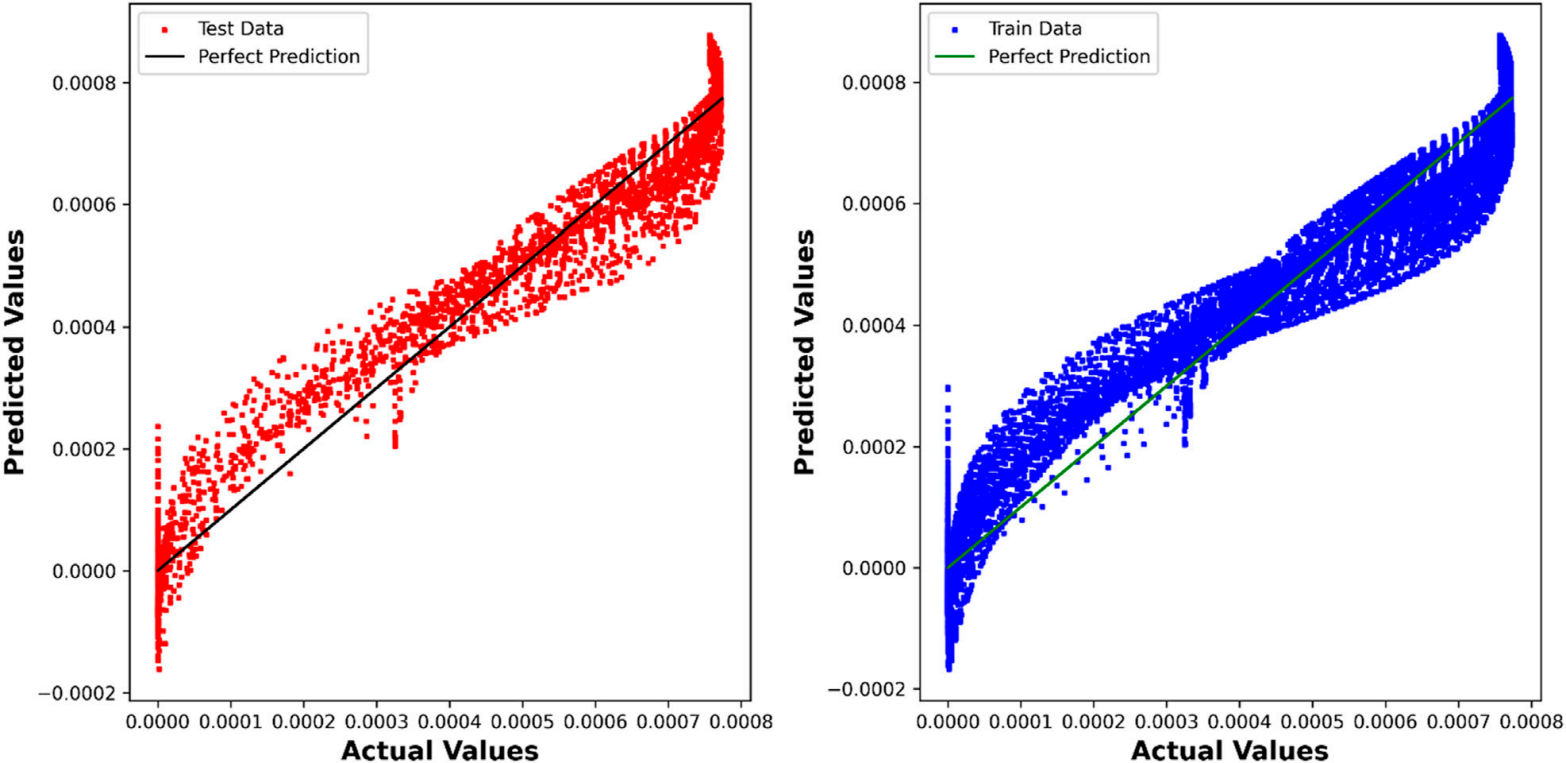
PAR model: Predicted values compared to True values.

While the PAR model demonstrates a slightly lower R^2^ score of 0.94652, its performance remains commendable, with competitive RMSE, MAE, and Max Error values. This indicates the model’s effectiveness in capturing relationships within the dataset, albeit with a nuanced trade-off between accuracy and complexity. In Figure 5, a visual comparison is presented, illustrating the disparities between the values predicted by the PAR model and the actual values.

**FIGURE 6:**
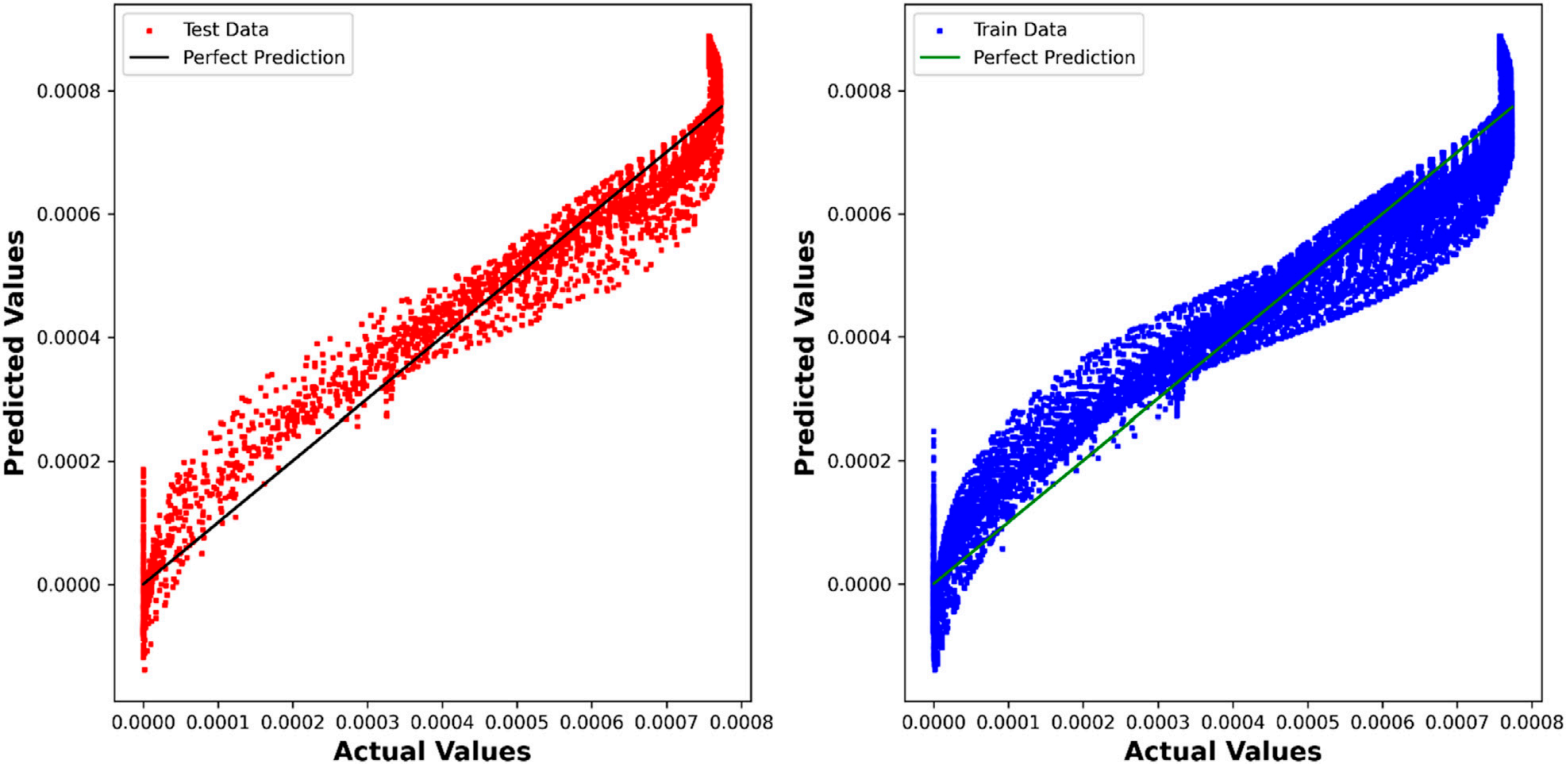
QPR model: Predicted values compared to True values.

The QPR model, designed to capture non-linear relationships, achieves a noteworthy R^2^ score of 0.95382. The model’s competitive RMSE, MAE, and Max Error values underscore its proficiency in accommodating the inherent complexities of the chemical engineering dataset. Figure 6 provides a visual representation, demonstrating the distinctions between the values forecasted by the QPR model and the factual values.

**FIGURE 7:**
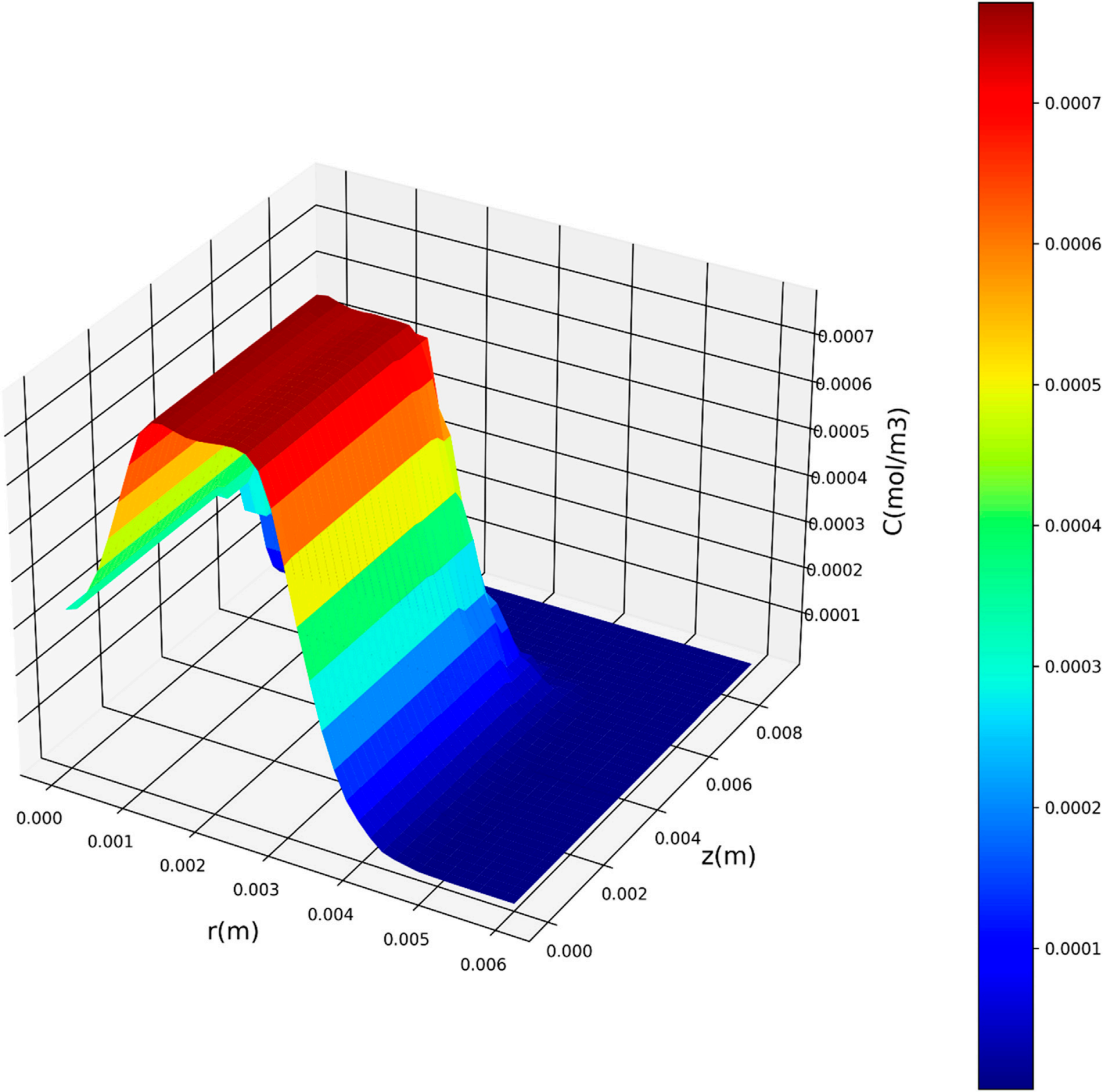
Three-dimensional representation of concentration with respect to r(m) and z(m) utilizing the DTR model.

**FIGURE 8:**
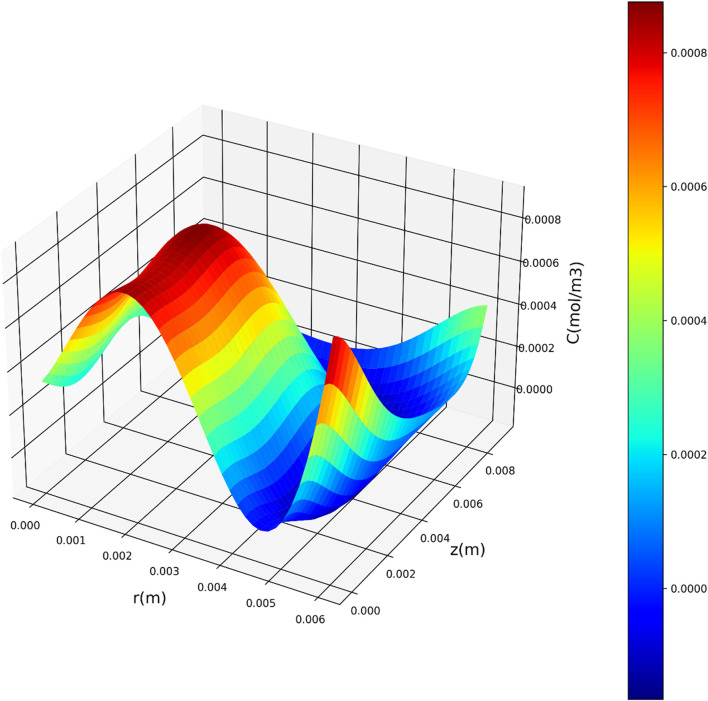
Three-dimensional representation of concentration with respect to r(m) and z(m) utilizing the PAR model.

**FIGURE 9:**
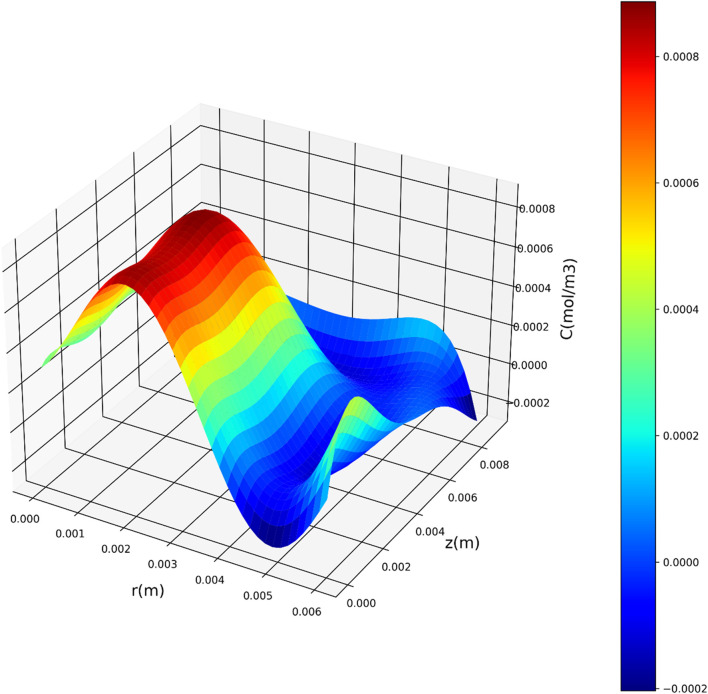
Three-dimensional representation of concentration with respect to r(m) and z(m) utilizing the QPR model.

Figures 7–9 present three-dimensional representations of concentration in relation to the variables *r*(m) and *z*(m), utilizing the three regression models. These visualizations offer a comprehensive view of how concentration varies across different values of *r*(m) and *z*(m) for each model. The change in drug concentration which has been obtained by the model could be attributed to the molecular diffusion occurring indie the polymeric matrix. Although both convective and diffusional mass transfer have been considered in the mass transfer model, the contribution of diffusion is significant and controls the release of drug from the carrier.

**FIGURE 10:**
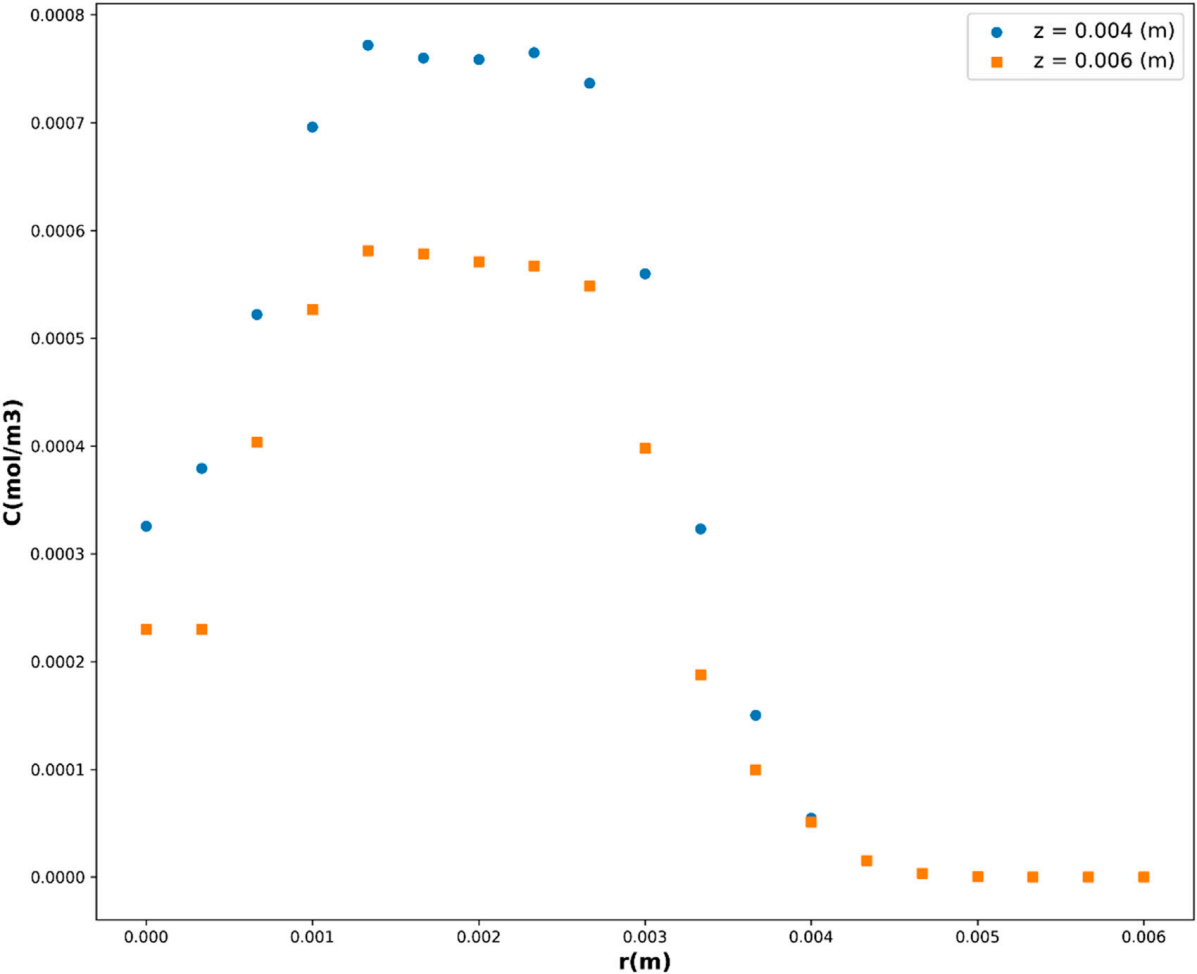
Concentration’s dependency on r.

**FIGURE 11:**
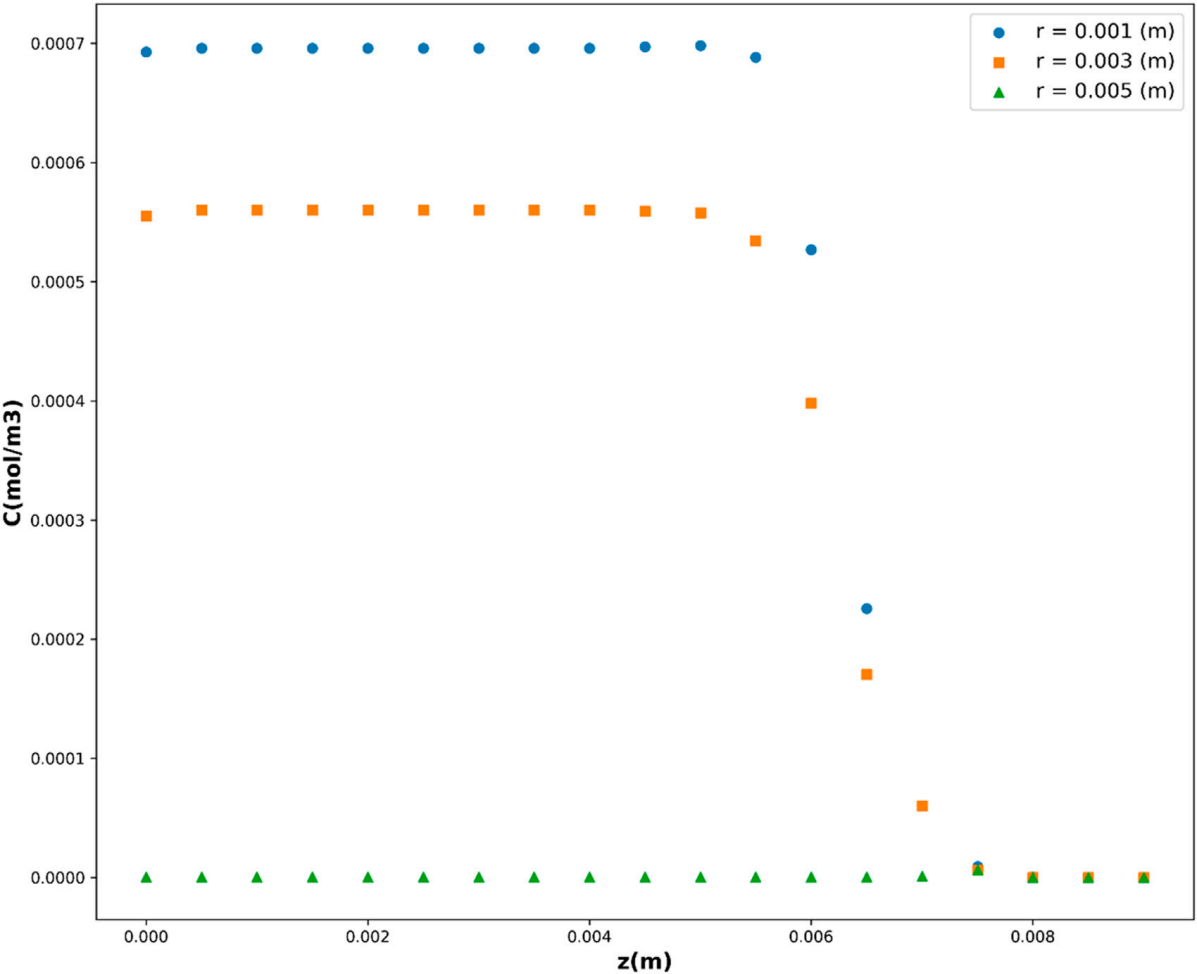
Concentration’s dependency on z.

Leveraging the DTR model, acknowledged as the top-performing model in this investigation, Figures 10, 11 depict the partial dependency of concentration on the variables *r*(m) and *z*(m), respectively. These visualizations provide insights into how changes in *r*(m) and *z*(m) influence the drug concentration, while keeping the other variable constant at multiple levels. This visualization provides a comprehensive representation of how the concentration varies across different combinations of the input variables, *r*(m) and *z*(m). The center of geometry is the drug where its concentration is the highest, while concentration declines beyond the center due to the diffusion as well as chemical reactions.

## 5 Conclusion

In conclusion, this paper has presented a rigorous evaluation of three distinct regression models, namely, Decision Tree Regression (DTR), Passive Aggressive Regression (PAR), and Quadratic Polynomial Regression (QPR), within the context of a dataset containing more than 15,000 data points. The dataset has been obtained from mass transfer simulation of drug release from a porous polymeric carrier. The input parameters, *r*(m) and *z*(m), were utilized to predict the output concentration (C) in mol/m³. The models underwent hyper-parameter optimization through Sequential Model-Based Optimization (SMBO), ensuring a meticulous exploration of the parameter space.

The results showcase the exceptional predictive capabilities of the Decision Tree Regression model, evidenced by a significant R^2^ score of 0.99887, a negligible RMSE of 9.0092E-06, a minute MAE of 3.51486E-06, and a maximum error of 6.87000E-05. Despite a slightly lower R2 score, the Passive Aggressive Regression model demonstrated commendable performance, while the Quadratic Polynomial Regression model showcased proficiency in capturing non-linear relationships within the dataset.

This comparative analysis not only provides valuable insights into the specific strengths and limitations of each regression model but also serves as a guide for practitioners in selecting an appropriate model tailored to the complexities of chemical engineering datasets. The incorporation of SMBO contributes to the robustness of the models, highlighting the significance of thoughtful hyper-parameter tuning in enhancing predictive accuracy. Overall, this research contributes to the ongoing discourse on regression model selection and optimization techniques in the domain of drug delivery, offering a foundation for further exploration and refinement in predictive modeling methodologies for design of advanced drug delivery systems.

## Data Availability

The original contributions presented in the study are included in the article/Supplementary material, further inquiries can be directed to the corresponding author.
